# Air pollution from natural and anthropic sources and male fertility

**DOI:** 10.1186/s12958-018-0430-2

**Published:** 2018-12-23

**Authors:** Joanna Jurewicz, Emila Dziewirska, Michał Radwan, Wojciech Hanke

**Affiliations:** 1 Department of Environmental Epidemiology, Nofer Institute of Occupational Medicine, 8 Teresy St, 91-362 Lodz, Poland; 2 Department of Gynecology and Reproduction, “Gameta” Hospital, 34/36 Rudzka St, 95-030 Rzgów, Poland; 3 Faculty of Health Sciences, The State University of Applied Sciences in Plock, 2 Dąbrowskiego Sq, 09-402 Płock, Poland

**Keywords:** Air pollution, Environmental exposure, Occupational exposure, Semen quality, Male fertility

## Abstract

Exposure to air pollution has been clearly associated with a range of adverse health effects, including reproductive toxicity. However, a limited amount of research has been conducted to examine the association between air pollution and male reproductive outcomes, specially semen quality. We performed a systematic review (up to March 2017) to assess the impact of environmental and occupational exposure to air pollution on semen quality. Epidemiological studies focusing on air pollution exposures and male reproduction were identified by a search of the PUBMED, MEDLINE, EBSCO and TOXNET literature bases. Twenty-two studies were included which assess the impact of air pollutants (PM_2.5_, PM_10_, SO_2_, NOx, O_3_, PAHs) on main semen parameters (sperm concentration, motility, morphology), CASA parameters, DNA fragmentation, sperm aneuploidy and the level of reproductive hormones. The number of studies found significant results supporting the evidence that air pollution may affect: DNA fragmentation, morphology and motility.

In summary, most studies concluded that outdoor air pollution affects at least one of the assessed semen parameters. However the diversity of air pollutants and semen parameters presented in the studies included in the review and different study design caused lack of consistency in results and difficulties in comparison.

## Introduction

During the past decades a possible degradation in human semen quality has been debated intensively and has become an important public health issue. A controversial review article of 61 studies analyzing sperm concentrations in fertile men and in men of unknown fertility published between 1938 and 1990 by Carlsen et al., 1992 showed a significant decrease in sperm concentrations (from 113 mln/ml to 66 mln/ml) and in semen volume (from 3.40 ml to 2.75 ml) [1]. Critics suggested that changing laboratory methods, statistical issues, heterogenicity of populations selected for studies (men of proven fertility or not, different geographical regions and ethnic groups), bias because of factors such as age and abstinence time or inherent variability of sperm counts might have affected the findings [2–4]. However more recent analysis gives further evidence for declining sperm quality. Swan et al., 2000 performed multivariate analysis of 101 studies from 1934 to 1996, taking into account many of the confounding factors, reported an even greater reduction in sperm concentration, indicating an annual decline of 1.5% in the USA compared with the 1% previously determined by Carlsen et al., 1992 [5].

Over time the World Health Organization has lowered the accepted values for normal semen parameters (count, motility and morphology) because in the last decades those parameters have consistently decreased even in healthy men [6]. It has been suggested that this decrease in semen quality is associated with the observed decrease in fertility [7].

This has raised new concerns about environmental factors such as exposure to pollutants or toxicants, and lifestyle factors such as smoking, heat, stress, obesity and sexual behaviours which might affect human fertility [8–11].

Air pollutants can be in the form of solid particles, liquid droplets, or gases. In addition, they may be natural or man-made. Sources of air pollution refer to the various locations, activities or factors which are responsible for the releasing of pollutants into the atmosphere. Particulate matter (PM) in the respirable range (PM_2.5_) is of particular interest, because it can carry multiple trace elements and polycyclic aromatic hydrocarbons (PAHs), a group of compounds that include several endocrine disruptors which may affect both the hypothalamic pituitary axis and testicular spermatogenesis and have the potential for causing sperm alterations [12, 13].

Ambient air pollution has been associated with a variety of health effects including cardiovascular [14] and respiratory diseases [15], adverse pregnancy outcome or impaired neurodevelopment in children [16]. However, a limited amount of research has been conducted to examine the association between air pollution and male reproductive outcomes, specifically semen quality.

The aim of this review was to assess current evidence regarding the impact of air pollution on male fertility.

## Materials and methods

Epidemiological studies focused on the exposure to air pollution and male fertility were identified by a search of the PubMed, Medline and Ebsco literature databases (before March 2017). The search combined terms referring to outdoor air pollution and male fertility. The combination of following key words was used: 1) referred to the exposure: exposure to air pollutants: PM_2.5_, PM_10_, SO_2_, NOx, O_3_, polycyclic aromatic hydrocarbons (PAHs); 2) referred to outcome: main semen parameters (sperm concentration, motility, morphology), CASA (Computer-Assisted Sperm Analysis) parameters (VAP (average path velocity), VSL (straight velocity), VCL (curvilinear velocity), ALH (amplitude of lateral displacement of the sperm head, LIN (linearity), BCF (beat-cross frequency), STR (path straightness), DNA fragmentation, sperm aneuploidy and the level of reproductive hormones.

From each study, the following information was abstracted: authors, the year published, the year studied, study design, population demographics, results, the main conclusions, exposure and methods used for its assessment (including biomarkers) and confounding factors. We included cohort, case-control and cross-sectional studies that analyzed the impact of outdoor air pollutants on male fertility in humans. The studies with exposure to most commonly assessed air pollutants: PM_2.5_, PM_10_, SO_2_, NOx, O_3_, PAHs were included. As just a few studies evaluated different air pollutants e.g. lead or cadmium those studies were not included in the present review. We excluded studies that analyzed the effects of air pollution on pregnancy outcome as well as those assessing the effects of lifestyle factors (smoking, alcohol consumption, caffeine intake). Also studies focused on animal research*,* in vitro studies and review papers were excluded. We limited the language only to English and we included only peer-review original articles.

Data were independently extracted by two investigators, who determined eligibility. Discrepancies were resolved by intervention of a third independent author. If multiple published reports from the same study were available, only the one with the most detailed information was included. All pertinent reports were retrieved and the relative reference lists were systematically searched in order to identify any potential additional studies that could be included.

## Results

In our initial search (up to March 2017) a total of 250 studies were retrieved in the initial electronic search. Of these, 210 were excluded by abstract because were focused on the animal research, were concentrated on molecule level or the study was a review according to the exclusion criteria described above, leaving 30 articles for inclusion in our analysis. Nine of the studies were excluded because the full text was not in English and because it was a review article or meta-analysis. The total of 22 articles were included. Remaining articles were excluded because did not assess outdoor air pollution (mainly indoor air pollution). Details of the studies included in the review are presented in Table 1.

**Table 1:** Details of the studies included in the review

Source	Location	Period	Study population	Age of men	Type of study	Exposure (range min-max)	Outcome
Environmental exposure							
Santi, et al. 2016 [25]	Italy, Modena	Nov 2014 to Feb 2015	406 men from the Clinical Pathology of the Nuovo Ospedale Civile Sant Agostino Estense (NOCSAE)	32.3 ± 5.2	Retrospective cohort study	PM10 (4–155 μg/m^3^)	Volume, concentration (× 10^6^/mL), total sperm number, typical/atypical forms (%),progressive motility (%), non-progressive motility (%), total motility (%), leucocytes
						PM2.5 (1–101 μg/m^3^)	
Wu, et al. 2016 [27]	China, Wuhan	March 2013 to Dec 2015	1759 men, partners of women undergoing assisted reproductive technology	34.4 ± 5.4	Retrospective cohort study	PM10 (67.2–197 μg/m^3^)	Concentration (× 10^6^/mL), sperm count, total motility (%), progressive motility (%),
						PM2,5 (27.3–172.4 μg/m^3^)	
Radwan, et al. 2016 [23]	Poland, Lodz	Jan 2008 to Apr 2011	327 men from infertility clinic	32.3 ± 4.4	Cross-sectional	PM10 (11.78–120.5 μg/m^3^)	Concentration (× 10^6^/mL), motility (%), sperm with abnormal morphology (%), DFI(%), HDS(%), CASA parameters: VSL, VCL, LIN; FSH, E_2_, T
						PM2.5 (7.99–93.89 μg/m^3^)	
						SO_2_ (9.12–167.9 μg/m^3^)	
						CO (0.15–1.85 μg/m^3^)	
						NO_x_ (2.17–215.14 μg/m^3^)	
Jurewicz, et al. 2015 [34]	Poland, Lodz	–	212 men from infertility clinic	22–57	Cross-sectional	PM10 (11.59–122.8 μg/m^3^)	Sperm aneuploidy, sperm concentration (× 10^6^/mL), total motility (%), abnormal morphology (%)
				32.25 ± 5.72		PM2,5 (7.99–95.22 μg/m^3^)	
						O_3_ (12.33–79.75 μg/m^3^)	
						SO_2_ (10.11–160.90 μg/m^3^)	
						CO (0.17–1.79 μg/m^3^)	
						NO_x_ (2.32–218.30 μg/m^3^)	
Radwan, et al. 2015 [35]	Poland, Lodz	Jan 2008 to Apr 2011	181 men from infertility clinic	32.1 ± 4.6	Cross-sectional	1 PAH metabolites in urine 1-OHP (0.04–2.03 μg/g creat)	Sperm aneuploidy, sperm concentration (× 10^6^/mL), total motility (%), normal sperm morphology (%),
Zhou, et al. 2014 [21]	China, Chongqing	2007	1346 men from family planning institutions	20–40	Cross-sectional	PM10 (66–160.5 μg/m^3^)	Volume, concentration (× 10^6^/mL), progressive motility (%), total motility, morphology (normal forms %), CASA sperm motility parameters (VCL, VSL, VAP, BCF, ALH, LIN, STR)
						SO_2_ (31-101 μg/m^3^)	
						NO_2_ (19.5–53.5 μg/m^3^)	
Jurewicz, et al. 2013 [24]	Poland, Lodz	–	277 men from infertility clinic	32 ± 4.6	Cross-sectional	1 PAH metabolite in urine 1-OHP (0.02–2.03 μg/g creat)	Volume, concentration (× 10^6^/mL), motility (%), atypical sperm (%),static sperm (%), CASA parameters: VAP, VSL, VCL, BCF, ALH, DFI (%)
Song, et al. 2013 [22]	China, Pearl River Delta	Jul 2010 to Aug 2011	53 men from infertility clinic		Cross-sectional	16 PAHs in blood	Concentration (× 10^6^/mL), volume, motility (grade A, grade B, grade C)
Han, et al. 2011 [31]	China, Chongqing	Dec 2007	232 men from Chongqing Family Planning Research Institute	31.89 ± 5.53	Cross-sectional	4 PAH metabolites in urine	Apoptotic marker (Annexin V^−^/PI^−^ spermatozoa %, Annexin V^+^/PI^−^ spermatozoa %, PI^+^ spermatozoa %, comet parameters (tail%, tail length, TDM)
						1-OHP (μg/g creatinine)	
						9-OHPh (μg/g creatinine)	
						2-OHFIu (μg/g creatinine)	
						2-OHNa (μg/g creatinine)	
Hammoud, et al. 2010 [20]	USA, Salt Lake City	2002 to 2007	1699 semen analyses and 877 inseminations	32.8 ± 6.57	Ecological study	PM2.5 (5-24 μg/m^3^)	Motility, concentration (×10^6^/mL), morphology (normal forms %)
Hansen, et al. 2010 [19]	USA, Wake County, Shelby County and Galveston County	2002 to 2004	228 men, partners of pregnant women	18–40	Cross-sectional	O_3_ (2–83.2 ppb)	Concentration (× 10^6^/mL), count, morphology (normal forms %), abnormal morphology (%), abnormal head (%), abnormal midsection (%), abnormal tail (%), cytoplasmic droplets (%), CMA (%), DFI (%)
						PM2.5 (2.1–62.7 μg/m^3^)	
Xia, et al. 2009a [28]	China, Nanjing	March 2004 to Jul 2007	513 infertile men and 273 fertile men as controls	28.65 ± 4.51 for infertile males and 29.32 ± 4.51 for controls	Cross-sectional	4 PAH metabolites in urine	Volume, concentration (×10^6^/mL), sperm number per ejaculum, sperm motility
						1-N (μg/g creat)	
						2-N(μg/g creat)	
						1-OHP (μg/g creat)	
						2-OHF(μg/g creat)	
Xia, et al. 2009b [29]	China, Nanjing	March 2004 to Jul 2007	542 men	No information -based on abstract	Cross-sectional	4 PAH metabolites in urine	Volume, concentration (× 10^6^/mL), sperm number per ejaculum, sperm motility
						1-N (μg/g creat)	
						2-N(μg/g creat)	
						1-OHP (μg/g creat)	
						2-OHF(μg/g creat)	
Rubes, et al. 2007 [32]	Czech, Teplice	Sep 1995 to Sep 1997	36 men	19–21	Longitudinal study	SO_2_ (μg/m^3^)	%DFI, *GSTM1* genotype
						NO_x_ (μg/m^3^)	
						PM10 (16.9–76.3 μg/m^3^)	
						PAH (21.4–221.9 ng/m^3^)	
Sokol, et al. 2006 [18]	USA, Los Angeles	Jan 1996 to Dec 1998	48 sperm donors from sperm donor bank	19–35 mean 25.3 ± 4.7	Retrospective cohort study	O_3_ (1.69–47.51 ppb)	Concentration (×10^6^/mL), motility (×10^6^)
						NO_2_ (9.04–79.80 ppb)	
						CO (0.37–3.86 ppm)	
						PM10 (6.84–101.88 μg/m^3^)	
Rubes, et al. 2005 [26]	Czech, Teplice	Sep 1995 to Sep 1997	36 men	19–21	Prospective cohort study	SO_2_ (μg/m^3^)	Count, concentration (×10^6^/mL), volume, motility (%), normal sperm head morphology (%), normal morphology (%), straight line velocity, curvilinear velocity, linearity, %DFI
						NO_x_ (μg/m^3^)	
						PM10 (μg/m^3^)	
						PAH (ng/m^3^)	
Selevan, et al. 2000 [17]	Czech, Teplice, Prachatice	1993 to 1994	408 men from Hygiene Station	18	Prospective cohort study	PM10 (3.1–832.0 μg/m^3^)	Semen volume, concentration (×10^6^/mL), total count, motility (%), total progressive, normal morphology (%), normal heads (%), VSL, VCL, LIN, sperm chromatin structure
						SO_2_ (1–697.9 μg/m^3^)	
						CO (0–5.50 mg/m^3^)	
						NO_x_ (0–367.20 μg/m^3^)	
Robbins, et al. 1999 [33]	Czech, Teplice, Prachatice		Subset of 32 men	No information -based on abstract	Prospective cohort study	No information-based on abstract	Sperm aneuploidy
Occupational exposure							
Calogero, et al. 2011 [37]	Italy	1.) 15June to 15July 2.) 1 to 31Jan	36 men working at motorway tollgates; 32 unexposed men	28–47 37.1 ± 5.5	Cross-sectional	NO_x_ (μg/m^3^)	LH, FSH, T, sperm concentration (×10^6^/mL), total count, total motility (%), progressive motility (%), normal forms (%), sperm chromatin integrity, DFI (%)
						SO_x_ (μg/m^3^)	
Boggia, et al. 2009 [38]	Italy	2000 to 2004	307 men working at motorway	23–57 mean 37.16	Cross-sectional	NO_2_ (μg/m^3^)	FSH, LH, T, sperm count, motility (%), morphology,
Guven, et al. 2008 [36]	Turkey	–	38 men working at motorway and 35 men working at office as a control group	35.2 ± 6.4 (study group) and 33.7 ± 6.7 (control group)	Cross-sectional	Traffic pollutants mainly the diesel	Concentration (×10^6^/mL), motility, morphology
De Rosa, et al. 2003 [39]	Italy	Jan2000 to Jan2002	85 men working at motorway and 85 control men randomly selected;	38.6 ± 0.8 (study group) and 39.6 ± 0.7 (control group)	Cross-sectional	CO (9-27 mg/m^3^)	FSH, LH, T, sperm count, volume, motility, morphology, sperm membrane function, forward progression, sperm kinetics: VSL, VCL, LIN, ALH,
						NO (58–398 mg/m^3^)	
						SO (9–27 μg/m^3^)	
						Pb (0.9–4.2 μg/m^3^)	

*PM10* particulate matter < 10 μm, *PM2,5* particulate matter < 2,5 μm, *O*_*3*_ ozone, *SO*_*x*_ sulphur oxides, *CO* carbon monoxide, *NO*_*x*_ nitrogen oxides, *PAH* polycyclic aromatic hydrocarbons, *1-OHP* 1-hydroxypyrene, *Pb* lead, Cd – cadmium, *1-N* 1-hydroxynapthalene, *2-N* 2-hydroxynapthalene, *1-OHP* 1-hydroxypyrene, *2-OHF* 2-hydroxyfluorene, *VCL* curvilinear velocity, *VSL* straight line velocity, *VAP* average path velocity, *BCF* beat cross frequency, *ALH* amplitude of lateral head displacements, *%DFI* percentage of sperm with fragmented DNA, *%HDS* high DNA stainability, *LIN* linearity, *STR* straightness, *grade A* sperm with progressive motility, *grade B* nonlinear motility, *grade c* non-progressive motility, *TDM* tail distributed moment, *%CMA* percentage of sperm chromatin maturity, *LH* luteinizing hormone, *FSH* follicle-stimulating hormone, *T* testosterone, *E*_*2*_ estradiol

## Summary of collected data

### Environmental exposure to air pollutants

#### Main semen parameters (motility, morphology, sperm concentration)

Thirteen studies examine the association between main semen parameters and environmental outdoor air pollution [17–29]. Three studies were performed in USA [18–20], five in China [21, 22, 27–29] and two in Poland [23, 24] and in Czech Republic [17, 26] and one in Italy [25]. In six of the presented studies the study population was recruited from general population [17, 19, 21, 22, 25, 26]. The level of PAHs were analyzed in four studies [22, 24, 28, 29] in urine [24, 28, 29] and blood [22].

In the study performed in Czech Republic the authors noticed that men exposed to air pollution were more likely to have lower percentage of motile sperm (β = − 8.12; 95%CI: -12.95, − 3.30) and lower percentage of sperm with normal morphology (β = − 0.84; 95%CI: -1.15, − 0.53) (fewer sperm with normal morphology or normal head shape) than were those lived in a city with less air pollution [17]. Later study among the same participants did not find the association between high air pollution and sperm concentration, volume, motility and morphology [26]. The Authors concluded that the inconsistency between studies could be due to differences in the exposures.

In the study in Los Angeles, California environmental exposure to ozone (O_3_) was associated with lower sperm concentration (*p* < 0.01) [18]. The ozone exposure was also associated with decrease in sperm concentration and count. Whereas exposure to PM_2.5_ increase the percentage of sperm cells with cytoplasmic drop and abnormal heads (but only in basic model without adjusting for potential confounding factors) in the study performed in United States by Hansen and co-workers (2010) [19]. Hammoud et al., 2010 in study conducted in Salt Lake City, Utah, found a negative association between PM_2.5_ and sperm motility and sperm head morphology (*p* = 0.010 and *p* = 0.044 respectively) [20].

In urban and rural areas in China the study performed among 1346 volunteers observed that the concentration of PM_10_, SO_2_ and NO_2_ were negatively associated with normal sperm morphology percentage (*p* < 0.001) [21]. The next study in China noticed a significant negative relation between semen motility and the concentration of PAHs (polycyclic aromatic hydrocarbons) in blood among 53 infertile volunteers (*p* < 0.01) [22]. The next study among 1759 men undergoing assisted reproductive technology procedures also performed in China found that exposure to PM_2.5_ was inversely associated with sperm concentration (β = − 0.20; (95%CI: -0.34, − 0.07) and count (β = − 0.22; 95%CI: -0.35, − 0.08) [27].

Santi et al., 2016 in Italian study observed that PM_2.5_ was directly related to total sperm number (*p* < 0.001). PM_10_ was directly related to both semen volume (0 < 0.001), and typical forms (p < 0.001), inversely related to atypical forms (p < 0.001), but related neither to sperm concentration (*p* = 0.430) nor to sperm motility [25]. Also Radwan et al., 2016 observed statistically significant association between abnormalities in sperm morphology and exposure to air pollutants (PM_10_, PM_2.5_, SO_2_, NO_X_, CO) (*p* = 0.0002, *p* = 0.0001, p = 0.0001, *p* = 0.01, p = 0.0001, respectively) [23]. Additionally exposure to PM_10_, PM_2.5,_ CO was negatively associated with the level of testosterone (*p* < 0.05) [23]. The next study performed in Poland among the same men from infertility clinic examined the association between a biomarker of exposure to polycyclic aromatic hydrocarbons (1-hydroksypyrene (1-OHP)) and semen quality [24]. A positive relation was found between the level of 1-OHP in urine and sperm neck abnormalities (*p* = 0.001) as well as the percentage of static sperm cells and the level of 1-OHP decreased semen volume and motile sperm cells (*p* = 0.018) [24]. Human studies among patients from infertility clinics in China showed that subjects with higher urinary concentrations of 1-OHP, 2-hydroxyfluorene (2-OHF) and sum PAH metabolites (assessed as tertiles) were more likely to have idiopathic male infertility (*p*-value for trend 0.034, 0.022 and 0.022, respectively) [28]. Higher idiopathic infertility risk was found in the group of idiopathic infertile subjects with abnormal semen quality when two groups of idiopathic infertile subjects with different semen quality [28]. In the next study by the same authors Xia et al., 2009b was found that men with higher 1-OHP (assessed as quintiles) were more likely to have below-reference sperm concentration and sperm number per ejaculum [29].

#### CASA parameters

The CASA parameters were assessed only in 5 studies [17, 21, 23, 24, 26] this is probably because of the fact that CASA has not been applied widely in field studies because the logistics of recording the sample promptly (to avoid degradation of sperm motility over time) and controlling the temperature precisely are challenging in the field studies [30]. Zhou and co-workers 2014 observed inverse associations between sperm VCL (curvilinear velocity) and VSL and the PM_10_, SO_2_, NO_2_ (*p* < 0.001) [21]. Whereas Selevan et al., 2000 did not demonstrate any consistent negative associations between the quality of sperm motion and periods of high air pollution [17]. Rubes et al., 2005 in the same study population also did not observe any statistically significant association [26]. No association between CASA parameters: VSL, VCL, LIN and exposure to PM_10_, PM_2.5_, SO_2_, NO_X_, CO [23] and the level of 1-OHP in urine [24] was also noticed the in the study performed in Poland.

#### DNA fragmentation

Seven studies assess the exposure to air pollution and sperm chromatin structure [17, 19, 23, 24, 26, 31, 32]. Two of them [24, 31] assess the exposure to polycyclic aromatic hydrocarbons, using biomarkers of exposure. In most of the studies SCSA method was used to evaluate sperm DNA, only Han et al., 2011 [31] used TUNEL method. Men exposed to air pollution in Teplice region (Czech Republic) had more sperm with abnormal chromatin than those lived in a Prachatice with less air pollution (*p* < 0.05) [17]. In the group of the same men from Teplice, high air pollution exposure was associated with increased sperm DNA fragmentation (β = 0.19; 95%CI: 0.02–0.36) [26]. The next study performed by the same authors found an evidence for a gene-environment interaction between glutathione-S transferase M1 (GSTM1) and air pollution (presumably c-PAHs) [32]. This study revealed a statistically significant association between GSTM1 null genotype and increased percentage of sperm with fragmented DNA (%DFI) (β = 0.309; 95% CI: 0.129, 0.489) [32]. Furthermore, GSTM1 null men also showed higher %DFI in response to exposure to intermittent air pollution (beta = 0.487; 95% CI: 0.243, 0.731) [32].

The association between urinary polycyclic aromatic hydrocarbon metabolites and sperm DNA damage was examined among 232 men from general population in China [31]. The increased urinary 2-hydroxynaphthalene (2-OHNa) levels were associated with increased comet parameters including the percentage of DNA in the tail (%tail), tail length and tail distribution (β = 13.26%; 95%CI: 7.97–18.55; β = 12.25; 95%CI: 0.01–24.52; β = 7.55; 95%CI: 1.28–18.83 respectively). Whereas the urinary level of 1-hydroxypyrene was associated only with increased tail% (β = 5.32; 95%CI: 0.47–10.17) [31]. In the study in Poland Radwan et al., 2016 found that exposure to PM_2.5_ and PM_10_ increase the percentage of cells with immature chromatin (HDS) (*p* = 0.002, *p* = 0.0001, respectively), but no the DNA fragmentation index (DFI) [23]. Whereas in the study in the same population urinary levels of 1-OHP was not associated with DNA fragmentation index in sperm (DFI) [24]. Also Hansen and co-worker 2010 did not observe any statistically significant relation between PM_2.5_ and O_3_ and DNA integrity and chromatin maturity [19].

#### Sperm aneuploidy

The first study which examined the association between exposure to air pollution and sperm aneuploidy was performed by Robbins et al., 1999 who collected a subset of samples (*n* = 32) from a larger epidemiological investigation of air pollution and reproductive health [33]. The sex chromosomal aneuploidy, YY, was found to be five-fold higher in sperm following periods of exposure to high air pollution (as indicated by SO_2_ levels = 196.9 mug/m3) as compared to low exposure (SO_2_ = 32.0 mug/m3) (IRR = 5.25, 95%CI: 2.5,11.0) [33].

The study with larger sample size of 212 men who were attending an infertility clinic for diagnostic purposes in Poland found positive associations between exposure to PM_2.5_ and disomy Y (*p* = 0.001), sex chromosome disomy (*p* = 0.05) and disomy 21 (*p* = 0.03). Exposure to PM_10_ was associated with disomy 21 (*p* = 0.02) [34]. Conversely, exposure to ozone, CO, SO_2_, and NOx did not affect sperm aneuploidy [34]. The study performed among the same study population observed that level of 1-OHP in urine increase the total sex-chromosome disomy (p = 0.03) and chromosome-18 disomy (p = 0.03) [35].

On the other hand Rubes et al., 2005 did not find any association between exposure to periods of high air pollution and total aneuploidy among young men from Teplice [26].

### Occupational exposure to air pollutants

#### Main semen parameters (motility, morphology, sperm concentration)

One of the study which investigate the effects of traffic pollutants, mainly the diesel exposure on semen parameters was performed in Turkey among 38 men working as toll collectors at motorways and 35 men working as office personnel [36]. The differences regarding the abnormal sperm count and motility were significant between the groups (*p* = 0.002 and *p* = 0.003, respectively). Similarly, the ratio of sperm cells with normal morphology was significantly lower in the study group than that in the control group (*p* = 0.001) [36]. The adverse role of traffic pollutants on male fertility was also investigated in the study among 36 men working at motorway tollgates and 32 unexposed healthy men [37]. Sperm concentration, total sperm count, total and progressive motility, and normal forms were significantly lower in these men compared with controls (*p* < 0.05) [37]. Also male workers, employed in a motorway company occupationally exposed to NO_2_ had a significant lower sperm total motility than in not exposed workers (p < 0.05) [38]. Total motility, forward progression were significantly lower in tollgate workers versus controls in the study performed in Italy (*p* < 0.0001) [39].

#### CASA parameters

Only one study assess the occupational exposure to air pollution and CASA parameters [39]. Motorway tollgate workers had significantly lower the CASA parameters: VSL, VCL, LIN, ALH (amplitude of lateral head displacements) compared with age-matched men living in the same area (p < 0.0001) [39].

#### DNA fragmentation

Motorway tollgate workers had a significantly higher percentage of spermatozoa with damaged chromatin and DNA fragmentation, a late sign of apoptosis, compared with controls (*p* < 0.001) in the study performed by Calogero et al., 2011 [37]. In this study the sperm DNA was evaluated using two methods: SCSA and TUNEL.

#### Level of reproductive hormones

Luteinizing hormone (LH), follicle-stimulating hormone (FSH), and testosterone (T) serum levels were within the normal range in tollgate workers compared to controls in the two studies performed in Italy [37, 39]. Serum levels of LH and FSH the study performed by Calogero et al., 2011 [37] were (2.9 ± 0.7 (1.9–4.5) IU/l and 4.2 ± 1.1 (1.9–7.1) IU/l for cases and 3.2 ± 1.1 (1.5–6.5) IU/l and 4.3 ± 1.5 (2.4–7.1) IU/l for controls respectively). Whereas in the second Italian study the levels of reproductive hormones were as follows: LH (IU/l) 2.8 ± 0.2 (0.7–8.9) for cases 2.8 ± 0.1 (0.9–5.4) for controls, FSH and serum testosterone (μg/l) 4.1 ± 0.3 (0.7–13.5) and 4.8 ± 0.2 (2.3–9.2) respectively for cases and 3.2 ± 0.2 (0.9–6.3) and 4.7 ± 0.2 (2.9–10.8) respectively for controls [39].

## Discussion

This review shows that air pollution (environmental and occupational) may affect semen quality. All the papers included in the review reported significant association with at least one of the examined semen parameters (Table 2). Number of studies found a significant results supporting the evidence that air pollution may affect: DNA fragmentation, morphology and motility.

**Table 2:** Exposure to air pollutants and semen quality

Outcome	Air pollution								
	PM10	PM2,5	Pb, Cd	NOx	SO_x_	PAHs	O_3_	CO	Ehoust partciles - diesel
Volume	Santi 2016 + ↑ Zhou 2014 - Rubes 2007 - Rubes 2005 -	Santi 2016 -		Zhou 2014 - Rubes 2007 - Rubes 2005 -	Zhou 2014 - Rubes 2007 - Rubes 2005 -	Song 2013 - Han 2011 - Rubes 2007 - Jurewicz 2013 + ↑			
Concentration (×10^6^/mL)	Santi 2016 - Zhou 2014 + ↑ Rubes 2007 - Rubes 2005 - Wu 2016 + ↓ Sokol 2006 -	Santi 2016- Wu 2016 + ↓ Hammound 2010 - Hansen 2010 -		Zhou 2014 - Rubes 2007 - Rubes 2005 - Sokol 2006 - Calogero 2011 + ↓	Zhou 2014 - Rubes 2007 - Rubes 2005 - Calogero 2011 + ↓	Song 2013 - Han 2011 - Rubes 2007 - Xia 2009b + ↓	Hansen 2010 - Sokol 2006 + ↓	Sokol 2006 -	
Sperm count	Santi 2016 - Sokol 2006 - Rubes 2005 - Wu 2006 + ↓	Santi 2016 + ↑ Wu 2016 + ↓ Hansen 2010 -		Sokol 2006 - Rubes 2005 - Calogero 2011 + ↓ Boggia 2009 -	Rubes 2005 - Calogero 2011 + ↓	Han 2011 - Xia 2009b + ↓	Hansen 2010 -	Sokol 2006 -	Guven 2008 + ↓
Morphology (% of sperm with normal morphology) (%)	Santi 2016 + ↑ Zhou 2014 + ↓ Rubes 2007 - Selevan 2000 + ↓ Radwan 2016 + ↓	Santi 2016 - Hammound 2010 - Hansen 2010 – Radwan 2016 + ↓		Zhou 2014 + ↓ Rubes 2007 - Selevan 2000 + ↓ Calogero 2011 + ↓ Boggia 2009 – Radwan 2016 + ↓	Zhou 2014 + ↓ Rubes 2007 - Selevan 2000 + ↓ Calogero 2011 + ↓ Radwan 2016 + ↓	Han 2011 – Rubes 2007 – Jurewicz 2013 + ↓	Hansen 2010 – Radwan 2016 + ↓	Selevan 2000 + ↓	Guven 2008 + ↓
Progressive motility (%)	Santi 2016 - Wu 2016 - Zhou 2014 - Selevan 2000 + ↓	Santi 2016 - Wu 2016 -		Zhou 2014 - Selevan 2000 + ↓ Calogero 2011 + ↓	Zhou 2014 - Selevan 2000 + ↓ Calogero 2011 + ↓			Selevan 2000 + ↓	
Total motility (%)	Santi 2016 – Wu 2016 - Zhou 2014 - Rubes 2007 – Sokol 2006 - Rubes 2005 - Selevan 2000 + ↓	Santi 2016 – Wu 2016 - Hammound 2010 + ↓	De Rosa 2003 + ↓	Zhou 2014 - Rubes 2007 - Sokol 2006 - Rubes 2005 - Selevan 2000 + ↓ Calogero 2011 + ↓ Boggia 2009 + ↓ De Rosa 2003 + ↓	Zhou 2014 - Rubes 2007 - Rubes 2005 -Selevan 2000 + ↓ Calogero 2011 + ↓ De Rosa 2003 + ↓	Song 2013 + Han 2011 - Rubes 2007 - Jurewicz 2013 + ↑	Sokol 2006 -	Sokol 2006 -Selevan 2000 + ↓ De Rosa 2003 + ↓	Guven 2008 + ↓
Sperm DNA damage	Rubes 2007 + ↑ Rubes 2005 + Zhou 2014 + ↑ Selevan 2000 + Rawdan 2016 + ↓	Hansen 2010 - Radwan 2016 -		Rubes 2007 + ↑ Rubes 2005 + Zhou 2014 + ↑ Selevan 2000 + Calogero 2011 + ↓ Radwan 2016 - De Rosa 2003 + ↓	Rubes 2007 + ↑ Rubes 2005 + Zhou 2014 + ↑ Selevan 2000 + Calogero 2011 + ↑ Radwan 2016 – De Rosa 2003 + ↓	Rubes 2007 + ↑ Rubes 2005 + Han 2011 + ↑ Jurewicz 2013 +	Hansen 2010 - Sokol 2006 + ↑	Selevan2000 + Rawdan 2016 -	
Sperm aneuploidy	Jurewicz 2015 + ↑	Jurewicz 2015 + ↑			Robbins 1999 + ↑	Radwan 2015 + ↑			
Reproductive hormones	Radwan 2016 + ↑	Radwan 2016 + ↑	De Rosa 2003 -	Calogero 2011 - Boggia 2009 - De Rosa 2003 -	Calogero 2011 - De Rosa 2003 -			De Rosa 2003 - Radwan 2016 + ↑	

*PM10* particulate matter < 10 μ3, *PM 2,5* particulate matter < 2,5 μm, *Pb* lead, *Cd* cadmium, *NO*_*x*_ nitrogen oxides, *SO*_*x*_ sulfur dioxide, *PAH* polycyclic aromatic hydrocarbons, *O*_*3*_ ozone, *CO* carbon monoxide

+ significant association; − non significant association; ↑ increase; ↓ decrease

Other review studies on exposure to air pollutants and male fertility also indicated the link between air pollution and sperm motility [40], sperm DNA fragmentation and morphology [41]. Deng et al., 2016 [42] in the meta-analyis found an evidence that ambient air polluation could alter sperm parameters resulting in infertility. Additionally several animal toxicological studies have provided evidence that exposure to air pollutants could damage the testicles and impact on sperm quality and fertility [43].

### Adjustment for confounders

Details of the potential risk factors are shown in Table 3. The results of the presented studies were adjusted for well-known confounders such as abstinence period (days before semen collection), age, smoking status and drinking. In some studies the smoking status was confirmed by the measure of the cotinine level in saliva or urine [23, 24, 34]. Several studies also considered the caffeine intake [17], season [17, 23, 24, 26, 34], temperature [18], BMI [19, 21] and ethnicity [18, 19]. Other factors were adjusted in some studies, such as vitamin [19], past diseases [23, 24, 34] and work posture [38].

**Table 3:** The results of the studies on air pollution and male fertility

Source	Exposure assessment	Outcome measured	Confounders	Statistical analysis	Main faindings	Limitations	Strengths
Santi, et al. 2016 [25]	PM10 (μg/m^3^)	Volume, concentration (× 10^6^/mL), total number, typical forms (%), atypical forms (%), progressive motility (%), non-progressive motility (%), total motility (%), leucocytes	Temperature, humidity	Bivariate and multivariate logistic regression models	PM2.5 was directly related to total sperm number (p = 0.001); PM10 was directly related to semen volume (p < 0.001) and typical forms (*p* < 0.001), inversely related to atypical forms (p < 0.001)	No information about smoking; Daily PM exposure registered through the monitoring network of the quality of the air for the province of Madena; the relationship between environmental PM and semen quality was evaluated irrespective of the life-style and risk factors of each men	The cohort is highly representative of the entire population; Choosing the coldest season of the year in this area (the possible negative influence of high ambient temp. on sperm quality was excluded);
	PM2.5 (μg/m^3^)						
Wu, et al. 2016 [27]	PM10 (μg/m^3^)	Concentration(×10^6^/mL), sperm count, total motility (%), progressive motility (%),	Age, BMI, ethnic, education, smoking, drinking, abstinence, season, average ambient temperature	Multivariate linear mixed models	PM2.5 was inversely associated with sperm concentration (β = −0.20; (95%CI: − 0.34, − 0.07) and sperm count (β = − 0.22; 95%CI: − 0.35, − 0.08); PM10 was inversely associated with sperm concentration and count (*p* < 0.05)	The used of ambient PM exposures as a proxy for individual exposures; No investigation of the association between exposure and sperm morphology; the results cannot be directly generalized to populations in which PM exposure levels are too low; Participants from infertility clinic	Big sample size; A wide concentration range of PM exposure; the use of IDW interpolation to more precisely estimate individual PM exposure; the non-linear exposure-response relationships
	PM2.5 (μg/m^3^)						
Radwan, et al. 2016 [23]	PM10	Concentration (× 10^6^/mL), motility (%), sperm with abnormal morphology (%), DFI(%), HDS(%), CASA parameters: VSL, VCL, LIN; FSH, E_2_, T	Age, smoking, temperature (men from 90 days), past disease, time of sexual abstinence, season	Multivariate linear regression	Exposure to air pollutants (PM10, PM2,5, SO_2_, NO_x_, CO) increased the abnormalities in sperm morphology (p = 0.0002, p = 0.0001, *p* = 0.0001, p = 0.01, *p* = 0.0001, respectively); Negative association were found between air pollutants and testosterone levels (p < 0.05); There were a positive associations between PM10 and PM2,5 and HDS (p = 0.002, p = 0.0001, respectively)	participants from an infertility clinic; single semen sample; exposure levels assessed by considering the ZIP code of each participants;	Assessment of many different semen parameters, reproductive hormones levels and sperm chromatin structure; detailed questionnaire information allowed for control confounding factors in the analysis; cotinine measured in saliva to verified smoking status;
	PM2.5						
	SO_2_						
	CO						
	NO_x_						
Jurewicz, et al. 2015 [34]	PM10 (μg/m^3^)	Sperm aneuploidy, sperm concentration (×10^6^/mL), total motility (%), abnormal morphology (%)	Age, smoking, drinking, temperature, season, past disease, abstinence interval, distance from monitoring station, concentration, motility, morphology, PM10, SO_2_	multivariate analysis	There was a positive association between exposure to PM2.5 and disomy Y (p = 0.001), sex chromosome disomy (p = 0.05), disomy of chromosome 21 (p = 0.03); xposure to PM10 was associated with disomy 21 (p = 0.02)	Exposure levels assessed by considering the ZIP code of each participants; participants from an infertility clinic; single semen sample;	Many confounders included to the analysis; cotinine measured in saliva to verified smoking status; detailed questionnaire information allowed for control confounding factors in the analysis,
	PM2.5 (μg/m^3^)						
	O_3_ (μg/m^3^)						
	SO_2_ (μg/m^3^)						
	CO (μg/m^3^)						
	NO_x_ (μg/m^3^)						
Radwan, et al. 2015 [35]	1 PAH metabolite in urine (1-OHP)	Sperm aneuploidy, sperm concentration (× 10^6^/mL), total motility (%), normal sperm morphology (%),	Abstinence, age, smoking, season, past disease,	Multivariate analysis	Positive associations between level of 1-OHP in urine and total sex-chromosome disomy (p = 0.03); An increase in the frequency of disomy 18 was related to the level of 1-OHP in urine (*p* = 0.03)	participants from an infertility clinic; single semen sample; one biomarker of PAHs exposure- 1-hydroksypyrene (1-OHP)	cotinine measured in saliva to verified smoking status; detailed questionnaire information allowed for control confounding factors in the analysis,
Zhou, et al. 2014 [21]	PM10 (μg/m^3^)	Volume, concentration (×10^6^/mL), progressive motility (%), total motility, morphology (normal forms %), CASA sperm motility parameters (VCL, VSL, VAP, BCF, ALH, LIN, STR)	Age, education, smoking, drinking, BMI, abstinence, season,	Multivariate regression models	PM10, SO_2_, NO_2_ were negatively associated with a normal sperm morphology percentage (p < 0.001); there were inverse associations between sperm VCL and VSL value and PM10, SO_2_, NO_2_ (*p* < 0.001); PM10 was positively associated with sperm concentration (*p* = 0.031)	Used of monitoring data for the study sites to measure ambient air pollution; the measurement and prediction of PM10, SO_2_, NO_2_ exposure were performed outside; the used only a single semen sample	Big sample size; Exposure to air pollutants in both urban and rural areas; The use of mean concentration of each pollutant during the 90 days before sampling; Many confounders including to the analyses
	SO_2_ (μg/m^3^)						
	NO_2_ (μg/m^3^)						
Jurewicz, et al. 2013 [24]	1 PAH metabolite in urine (1-OHP)	Volume, concentration (×10^6^/mL), motility (%), atypical sperm (%),static sperm (%), DFI (%), CASA parameters: VAP, VSL, VCL, BCF, ALH,	Age, smoking, past disease, season, sexual abstinence,	Multivariate regression models	A positive associations were observed between the level of 1-OHP in urine and sperm neck abnormalities (p = 0.001) and a negative between the semen volume (*p* = 0.014), %motility (p = 0.0001) and %static sperm (p = 0.018);	Participants from an infertility clinic; single urine and semen sample; one biomarker of PAHs exposure- 1-hydroksypyrene (1-OHP)	cotinine measured in saliva to verified smoking status; detailed questionnaire information allowed for control confounding factors in the analysis,
Song, et al. 2013 [22]	16 PAHs in blood	Concentration (× 10^6^/mL), volume, motility (grade A, grade B, grade C)	–	The Pearson correlation analysis	Significant correlations between PAHs in blood and semen motility were observed (p < 0.01)	Small sample size; participants from infertility clinic; No investigation of the association between PAH exposure and semen morphology;	16 PAH metabolites as a biomarkers of exposure to PAH;
Han, et al. 2011 [31]	4 PAH metabolites in urine	Apoptotic marker (Annexin V^−^/PI^−^ spermatozoa %, Annexin V^+^/PI^−^ spermatozoa %, PI^+^ spermatozoa %, comet parameters (tail%, tail length, TDM)	Age, BMI, abstinence, smoking, drinking, grilled and smoked foods ingestions	Multivariate regression models	2-OHNa levels were associated with increased comet parameters including tail% (β = 13.26% per log unit 2-OHNa; 95%CI: 7.97–18.55), tail length (β = 12.25; 95%CI: 0.01–24.52) and tail distribution (β = 7.55; 95%CI: 1.28–18.83); 1-OHP was associated with increased tail% (β = 5.32; 95%CI: 0.47–10.17); urinary PAH metabolites were associated with decreased vital Annexin V negative sperm count;	Single urine sample; the use of biomarkers didn’t allow for determination of primary exposure sources;	Urinary PAH metabolites as biomarkers of PAH exposure; Assessing 4 metabolites individually; Participants recruited only during the winter when air pollution is higher); many confounders including in the analyses;
	1-OHP						
	9-OHPh						
	2-OHFIu						
	2-OHNa						
Hammoud, et al. 2010 [20]	PM2.5 (μg/m^3^)	Motility, concentration (×10^6^/mL), morphology (normal forms %)	Temperature, season	Multivariate regression models	PM2.5 was negatively associated with sperm motility 2 months and 3 months following the recording of the PM2.5 values (p = 0.010 and p = 0.044, respectively)	Participants from andrology laboratory; occupational exposure didn’t include in the analysis	Repeated semen samples; big sample size; The analyses of daily levels of PM2.5 over the 5 years;
Hansen, et al. 2010 [19]	O_3_ (ppb)	Concentration (× 10^6^/mL), count, morphology (normal forms %), abnormal morphology (%), abnormal head (%), abnormal midsection (%), abnormal tail (%), cytoplasmic droplets (%), CMA (%), DFI (%)	Age, abstinence, education levels, smoking, season, temperature	Multivariate regression models	Exposures to O_3_ or PM2.5 at least below the current National Ambient Air Quality Standards were not associated with decrements in sperm outcomes.	Small sample size; single semen sample;	Many confounders including in the analysis;
	PM2.5 (μg/m^3^)						
Xia, et al. 2009a [28]	4 PAH metabolites in urine	Volume, concentration (×10^6^/mL), sperm number per ejaculum, sperm motility	Age, abstinence time,	Logistic regression analysis	Men with higher urinary concentrations of 1-OHP, 2-OHP and Sum PAH metabolites were more likely to have idiopathic male infertility (p for trend = 0.034)	Single semen sample; No investigation of the association between PAH exposure and semen morphology;	Big sample size; Urinary PAH metabolites as biomarkers of PAH exposure; men with idiopathic infertility were divided into ‘normal’ and ‘abnormal’ semen quality group
	1-N						
	2-N						
	1-OHP						
	2-OHF						
Xia, et al. 2009b [29]	4 PAH metabolites in urine	Volume, concentration (× 10^6^/mL), sperm number per ejaculum, sperm motility	No information-based on abstract	No information-based on abstract	Men with higher 1-OHP (assessed as quintiles) were likely to have below-reference sperm concentration and sperm number per ejaculum.	No information-based on abstract	No information-based on abstract
	1-N						
	2-N						
	1-OHP						
	2-OHF						
Rubes, et al. 2007 [32]	SO_2_ (μg/m^3^)	%DFI *GSTM1* genotype	Smoking	Mixed models	Association between *GSTM1* null genotype and increased %DFI (beta = 0.309; 95% CI: 0.129, 0.489). Furthermore, GSTM1 null men also showed higher %DFI in response to exposure to intermittent high air pollution (beta = 0.487; 95% CI: 0.243, 0.731)	Small sample size; Single semen sample; only one confounder included to the analysis;	Novel evidence for a gene-environment interaction between *GSTM1* and air pollution;
	NO_X_ (μg/m^3^)						
	PM10 (μg/m^3^)						
	PAH (ng/m^3^)						
Sokol, et al. 2006 [18]	O_3_ (ppb)	Concentration (×10^6^/mL), motility (×10^6^)	Temperature, seasonality, age of donation, date of birth	Linear mixed-effects model	Negative association between ozone and sperm concentration (p < 0.01);	Small sample size; No investigation of the association between air pollution exposure and semen morphology;	Repeated semen samples;
	NO_2_ (ppb)						
	CO (ppm)						
	PM10 (μg/m^3^)						
Rubes, et al. 2005 [26]	SO_2_ (μg/m^3^)	Count, concentration (× 10^6^/mL), volume, motility (%), normal sperm head morphology (%), normal morphology (%), straight line velocity, curvilinear velocity, linearity, DFI%	Smoking, drinking, caffeine, abstinence, fever, briefs,	Mixed models for repeated measures	Significant association between high air pollution and %DFI (β = 0.19; 95%CI: 0.02–0.36); Other semen measures were not associated with air pollution;	Small sample size;	Urine sample were assayed for cotinine to confirm self-reported smoking status; Blood sample was collected for analysis of lead, mercury and cadmium as an indication of possible exposure to metals; Air pollution categorized as low and high; semen samples classify as a “winter” or “summer” sample
	NO_x_ (μg/m^3^)						
	PM10 (μg/m^3^)						
	PAH (ng/ m^3^)						
Selevan, et al. 2000 [17]	PM10 (μg/m^3^)	Semen volume, concentration (×10^6^/mL), total count, motility (%), total progressive, normal morphology (%), normal heads (%), VSL, VCL, LIN, sperm chromatin structure	Abstinence, wearing briefs, caffeine, high fever, work and hobbies with metal, work and hobbies with solvents, season	Multivariate regression models	Significant associations between medium air pollution and %motile sperm (β = −8.12; 95%CI: −12.95, −3.30); between medium air pollution and progressive motility (β = −0.15; 95%CI: −0.30, −0.01); Negative association between medium and high air pollution and %normal morphology (β = − 0.42; 95%CI: − 0.69, − 0.14 and β = − 0.84; 95%CI: − 1.15, − 0.53); High air pollution was associated with parameters of sperm motion – VSL, VCL, LIN (p < 0,005)	Single semen sample; Data about air pollution from the air monitoring program;	Air pollution categorized as low, medium and high; Many confounders included to the analysis
	SO_2_ (μg/m^3^)						
	CO (mg/m^3^)						
	NO_x_ (μg/m^3^)						
Robbins, et al. 1999 [33]	SO2	aneuploidy	Alcohol, caffeine intake, fever, laboratory variables	Poisson regression modeling	The sex chromosomal aneuploidy YY was associated with exposure to high air pollution IRR = 5.25, 95%CI: 2.5,11.0	No information-based on abstract	No information-based on abstract
Occupational exposure							
Calogero, et al. 2011 [37]	NO_x_ (μg/m^3^)	LH, FSH, T, sperm concentration (×10^6^/mL), total sperm count, total motility, progressive motility (%), normal form (%), sperm chromatin integrity; DFI (%)	Age, length of occupational exposure, smoking	Multivariate regression models;	Motorway tollgate workers had a significantly higher %spermatozoa with damage chromatin (p < 0.001) compared with controls, likewise the %spermatozoa with fragmented DNA; late sign of apoptosis was also significantly higher in tollgate workers (*p* < 0.001); sperm concentration, total sperm count, total and progressive motility, and normal form were significantly lower in tollgate workers compared with controls (p < 0.05)	Small sample size;	blood levels of MHb, SHb, COHb, Pb as a biological biomarkers of environmental pollution; the air pollution was measured with specific analyzers 24 h/day for 30 days during summer;
	SO_x_ (μg/m^3^)						
Boggia, et al. 2009 [38]	NO_2_ (μg/m^3^)	FSH, LH, T, sperm count, motility, morphology,	Fuel combustion gases		Negative association between men occupationally exposed to NO_2_ and total motility (p < 0.05)	No individual estimations of exposure;	Cross-sectional design
Guven, et al. 2008 [36]	diesel	Concentration (×10^6^/mL), motility, morphology	–	student’s t-test, Mann-Whitney *U*-test;	The differences regarding the abnormal sperm count and motility were significant between the study group and control group (p = 0.002 and p = 0.003, respectively); the ratio of sperm cells with normal morphology was significantly lower in the study group (p = 0.001)	Small sample size; No individual estimations of exposure;	Cross-sectional design
De Rosa, et al. 2003 [39]	CO (mg/m^3^)	FSH, LH, T, sperm count, volume, motility, morphology, sperm membrane function, forward progression, sperm kinetics: VSL, VCL, LIN, ALH,		student’s t-test; linear regression analysis	Total motility, forward progression, functional test and sperm kinetic were significantly lower in tollgate workers vs. controls (p < 0.0001)	Small sample size; No individual estimations of exposure	blood levels of MHb, SHb, COHb, Pb, Zn as a biological biomarkers of environmental pollution;
	NO (mg/m^3^)						
	SO (μg/m^3^)						
	Pb (μg/m^3^)						

*PM10* particulate matter < 10 μm, *PM2,5* particulate matter < 2,5 μm, *O*_*3*_ ozone, *SO*_*x*_ sulphur oxides, *CO* carbon monoxide, *NO*_*x*_ nitrogen oxides, *PAH* polycyclic aromatic hydrocarbons, *1-OHP* 1-hydroxypyrene, *Pb* lead, Cd – cadmium, *1-N* 1-hydroxynapthalene, *2-N* 2-hydroxynapthalene, *1-OHP* 1-hydroxypyrene, *2-OHF* 2-hydroxyfluorene, *VCL* curvilinear velocity, *VSL* straight line velocity, *VAP* average path velocity, *BCF* beat cross frequency, *ALH* amplitude of lateral head displacements, *%DFI* percentage of sperm with fragmented DNA, *%HDS* high DNA stainability, *LIN* linearity, *STR* straightness, *grade A* sperm with progressive motility, *grade B* nonlinear motility, *grade c* non-progressive motility, *TDM* tail distributed moment, *%CMA* percentage of sperm chromatin maturity, *LH* luteinizing hormone, *FSH* follicle-stimulating hormone, *T* testosterone, *E*_*2*_ estradiol, *MHb* methahaemoglobin, *SHb* sulphahaemoglobin, *COHb* carboxyhaemoglobin, *IDW interpolation* inverse distance weighting (Shepard’s method)

The major potential confounders in studies of exposure to air pollution and semen quality are well-known and most studies at least try to assess them. The concern is generally in the factors which are unmeasured such as stress or family support. Although available confounders were taken into account in the statistical analysis there is still possibility that residual or unmeasured confounding factors partly contributed to the observed association.

### Assessment of exposure

In most of the presented studies the exposure assessment was based on the information from monitoring stations for a specific period of time (90 days) before semen sampling (Table 1). Arithmetic mean for period of 90 days before semen collection was calculated as indicator of exposure. The process of spermatogenesis involves a series of complex steps (stem cell replication, meiosis, and spermiogenesis) over approximately 74 days in humans [44]. An exposure period of approximately 90 days is generally accepted as being of sufficient duration for detecting effects on any stage of spermatogeneis when using semen measures as the biologic end points [17].

The biomarkers of exposure was used in 6 of the presented studies. One of those studies were conducted in occupational settings and measured lead in the blood and methemoglobin as a marker of NO_2_, sulphemoglobin for SO_2_, carboxyhemoglobin and zinc propophyrin. In case of environmental exposure PAH metabolites were measured in urine in five studies [22, 24, 28, 29, 31]. Han et al., 2011 [31] and Xia et al., 2009a [28], Xia et al., 2009b [29] have assessed urinary level of four PAH metabolites whereas Jurewicz et al., 2013 [24] assessed only 1-hydroksypirene (1-OHP). Song et al., 2013 measured 16 PAHs levels in blood and semen [22].

### Differences in the results between studies

There are possibly numerous factors contributing to the divergent results between the studies. The various endpoints of semen quality (sperm concentration, motility, morphology, sperm DNA damage, sperm aneuploidy) used may be a possible explanation for the different study results. The use of different biomarkers to ascertain exposure or the exposure estimation based on the information from the monitoring station may have some bearing on the statistical association. Also different level of exposure to air pollutants may impact on the differences between studies. The choice of covariates for statistical models may also impact the results. Further issue is a possibility of concomitant exposure to other environmental or occupational factors which may also have an impact on semen quality. Other potential explanations for the differences among studies include the type and timing of exposure, dose, measurement of the exposure or an outcome.

### Biological mechanism

A limited number of animal toxicologic studies have provided preliminary evidence of associations between exposure to air pollutants and semen quality outcomes. Associations have been observed between total air pollution and reduced daily sperm production in mice and rats receiving in utero or prenatal exposure to total diesel exhaust and filtered exhaust [45]. The biological mechanisms linking ambient air pollution to decreased sperm quality have yet to be determined. Sokol et al., 2006 identified several possible mechanisms, including O_3_-induced oxidative stress, inflammatory reactions, and the induction of the formation of circulating toxic species [18]. Rubes et al., 2007 concluded that the reactive metabolites of PM_10_ can reach the testes and react with sperm DNA to form adducts; this toxic effect occurs in late spermatogenesis, when there are no repair mechanism to correct it, resulting in increased DNA fragmentation [32]. Additionally Hammound et al., 2010 suggest that PM_2.5_ could act as an endocrine disruptor affecting late synthesis of proteins necessary for sperm motility [20]. Additionally the reactive oxygen species damage the integrity of DNA in the sperm nucleus which may affect sperm count and motility [46, 47].

Rubes et al., 2007 found that men who are homozygous null for GSTM1 have lower capacity to detoxify reactive metabolites of carcinogenic polycyclic aromatic hydrocarbons and are consequently more susceptible to the effects of air pollution on sperm chromatin [32]. Also polymorphism in other repair genes (*XRCC1, XPD6, XPD23*) and observe an association with high or medium DNA sperm damage [48].

### Strength and limitations of the studies

The studies presented in this review are mostly well-design and adjusted for potential confounders (Table 3).

The limitation is the exposure assessment approach based on the information regarding air pollution from monitoring stations in the limitation in most of the reviewed studies. This is incapable of providing truly individual estimates of exposure. Individual’s precise exposure to any component of air pollution would be expected to depend on his location, activity patterns and the weather conditions. Additionally in most of the studies single semen samples were collected. Only Sokol et al., 2006 [18] and Hammound et al., 2010 [20] collected multiple semen samples, which is relevant considering the known high intraindividual variability in semen quality. Also in most of the studies there is no information about co-exposure. The next limitation arise from the fact that in most of the selected studies assessing the environmental exposure examined the semen samples among men from infertility clinic.

## Conclusions

In conclusion we have found that exposure to air pollution may affect semen quality, especially sperm DNA damage, morphology and motility. The diversity of the semen parameters used in the studies and different approach in exposure assessment made the comparison of the results difficult. Future research should use a better characterization of exposure models in order to validate the effects of air pollution on human sperm. Prospective studies in well-defined cohorts of men in various populations are needed to evaluate the potential effect of air pollution on male reproductive health. These studies should take into account other factors which may interfere with male reproductive health. Future studies should incorporate different seasons to generate a more accurate and full assessment of adverse effect of air pollution on male fertility.

## Abbreviations

%DFI: percentage of sperm with fragmented DNA
%tail: percentage of DNA in the tail
1-OHP: 1-hydroxypyrene
2-OHF: 2-hydroxyfluorene
2-OHNa: 2-hydroxynaphthalene
ALH: amplitude of lateral head displacements
FSH: follicle-stimulating hormone
GSTM1: glutathione-S transferase M1
HDS: high DNA stainability
LH: luteinizing hormone
LIN: linearity
NOx: nitrogen oxides
O_3_: ozone
PAHs: polycyclic aromatic hydrocarbons
PM: particulate matter
PM_10_: particulate matter < 10 μm
PM_2.5_: particulate matter < 2.5 μm
SO_2_: sulphur dioxide
VCL: curvilinear velocity
VSL: straight line velocity
